# Quantitative analysis of an engineered CO_2_-fixing *Escherichia coli* reveals great potential of heterotrophic CO_2_ fixation

**DOI:** 10.1186/s13068-015-0268-1

**Published:** 2015-06-18

**Authors:** Fuyu Gong, Guoxia Liu, Xiaoyun Zhai, Jie Zhou, Zhen Cai, Yin Li

**Affiliations:** CAS Key Laboratory of Microbial Physiological and Metabolic Engineering, Institute of Microbiology, Chinese Academy of Sciences, No. 1 West Beichen Road Chaoyang District, Beijing, 100101 China; University of the Chinese Academy of Sciences, Beijing, China

**Keywords:** Carbon fixation, CO_2_-fixation rate, Heterotrophic microbe, Carbonic anhydrase, Rubisco

## Abstract

**Background:**

Production of fuels from the abundant and wasteful CO_2_ is a promising approach to reduce carbon emission and consumption of fossil fuels. Autotrophic microbes naturally assimilate CO_2_ using energy from light, hydrogen, and/or sulfur. However, their slow growth rates call for investigation of the possibility of heterotrophic CO_2_ fixation. Although preliminary research has suggested that CO_2_ fixation in heterotrophic microbes is feasible after incorporation of a CO_2_-fixing bypass into the central carbon metabolic pathway, it remains unclear how much and how efficient that CO_2_ can be fixed by a heterotrophic microbe.

**Results:**

A simple metabolic flux index was developed to indicate the relative strength of the CO_2_-fixation flux. When two sequential enzymes of the cyanobacterial Calvin cycle were incorporated into an *E. coli* strain, the flux of the CO_2_-fixing bypass pathway accounts for 13 % of that of the central carbon metabolic pathway. The value was increased to 17 % when the carbonic anhydrase involved in the cyanobacterial carbon concentrating mechanism was introduced, indicating that low intracellular CO_2_ concentration is one limiting factor for CO_2_ fixation in *E. coli*. The engineered CO_2_-fixing *E. coli* with carbonic anhydrase was able to fix CO_2_ at a rate of 19.6 mg CO_2_ L^−1^ h^−1^ or the specific rate of 22.5 mg CO_2_ g DCW^−1^ h^−1^. This CO_2_-fixation rate is comparable with the reported rates of 14 autotrophic cyanobacteria and algae (10.5–147.0 mg CO_2_ L^−1^ h^−1^ or the specific rates of 3.5–23.7 mg CO_2_ g DCW^−1^ h^−1^).

**Conclusions:**

The ability of CO_2_ fixation was created and improved in *E. coli* by incorporating partial cyanobacterial Calvin cycle and carbon concentrating mechanism, respectively. Quantitative analysis revealed that the CO_2_-fixation rate of this strain is comparable with that of the autotrophic cyanobacteria and algae, demonstrating great potential of heterotrophic CO_2_ fixation.

**Electronic supplementary material:**

The online version of this article (doi:10.1186/s13068-015-0268-1) contains supplementary material, which is available to authorized users.

## Background

The wasteful greenhouse gas carbon dioxide (CO_2_) is a potential raw material for production of chemicals and fuels [1]. To this end, energy input is required since the carbon in CO_2_ is in its highest oxidation state. During the past 5 years, a variety of chemicals including ethanol [2–4], *n*-butanol [5–8], acetone [9], isobutyraldehyde [7], lactic acid [10–12], isoprene [13], 1,2-propanediol [14], methane [15], and biodiesel [16, 17] have been produced from CO_2_ by engineered autotrophic microbes such as cyanobacteria and algae, using light as the energy resource. Apart from the light, autotrophic microbes can also use hydrogen and/or sulfur as the energy source for CO_2_ assimilation under mild conditions [18].

Heterotrophic microbes usually do not assimilate CO_2_ through the central metabolism. Recent studies indicated that incorporation of several steps of a natural carbon fixation pathway into a heterotrophic microbe may create a CO_2_-fixing bypass pathway which enables the host to assimilate CO_2_ at the expense of carbohydrates. Examples include introduction of two enzymes of Calvin cycle into *Escherichia coli* and *Saccharomyces cerevisiae*, which resulted in enhanced CO_2_ recycling in an air-tight fermentor [19] and an increased ethanol yield [20], respectively.

Although these preliminary data suggested that heterotrophic CO_2_-fixation is feasible, little is done to quantitatively analyze and evaluate the process. To date, simple approaches capable of evaluating the CO_2_ flux in heterotrophic microbes are still lacking, since the metabolites of the CO_2_-fixing bypass pathway are indistinguishable from those of the central metabolic pathway. Due to lack of quantitative analysis, it remains unclear where the bottleneck for heterotrophic CO_2_-fixation is and whether the rate of heterotrophic CO_2_-fixation is higher, lower, or comparable with that of autotrophic CO_2_-fixation.

The aim of this study was to address the above issues through a quantitative and comprehensive analysis of the heterotrophic CO_2_-fixation process. To evaluate the strength of CO_2_ flux, a metabolic flux index, MFI_h-CO2_, was developed to indicate the metabolic flux ratio between the CO_2_-fixing bypass pathway and the central carbon metabolic pathway. The MFI_h-CO2_ was determined by addition of ^13^C-labeled sodium bicarbonate into the culture medium, followed by quantification of the isotropic-labeled and unlabeled forms of one intracellular metabolite by liquid chromatography–mass spectrometry/mass spectrometry (LC-MS/MS). Comparison of MFI_h-CO2_ values of several engineered CO_2_-fixing *E. coli* strains led to identification of the rate-limiting steps of heterotrophic CO_2_ fixation. The strain with the highest MFI_h-CO2_ value was aerobically cultivated in minimal medium supplemented with xylose in a chamber filled with 5 % CO_2_. The mass of fixed CO_2_ per liter culture of this strain per hour was calculated by the mass balance of carbon. The CO_2_-fixation rate in *E. coli* was then compared with those of several autotrophic microbes to evaluate the potential of heterotrophic CO_2_ fixation.

## Results

### Development of a metabolic flux index, MFI_h-CO2_, for relative quantification of heterotrophic CO_2_ fixation

It is costly and time-consuming to determine the absolute metabolic flux of CO_2_ fixation by quantifying every isotropic-labeled metabolite upon the feed of ^13^CO_2_ during cultivation. As the metabolic flux of the central metabolism for a given strain is quite stable, the relative metabolic flux of the CO_2_-fixing bypass pathway over that of the central carbon metabolic pathway may give a quantitative understanding on the efficiency of CO_2_ fixation. This relative value is then termed as the metabolic flux index of the heterotrophic CO_2_-fixation pathway, MFI_h-CO2_. At the conjunction of the CO_2_-fixing bypass pathway and the central pathway, the metabolite generated by the two pathways can be differentiated by using ^13^C-labeled CO_2_ and unlabeled sugar. The amount of the labeled and unlabeled forms of the joint metabolite can be determined and used to calculate the metabolic flux ratio of the two pathways to obtain the MFI_h-CO2_ value.

**Fig. 1:**
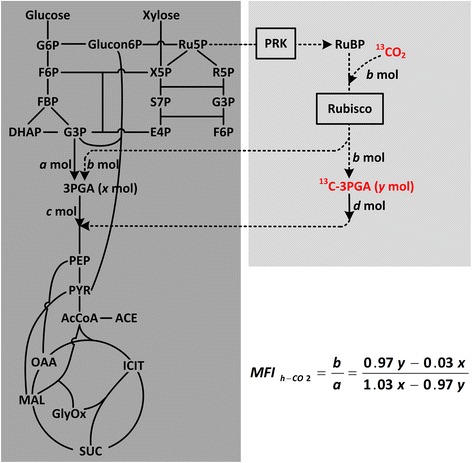
Metabolic pathway of a CO_2_-fixing *E. coli*. The central carbon metabolic pathway is shaded in *dark gray*, while the introduced CO_2_-fixation bypass pathway composed of PRK and Rubisco is shaded in *light gray*. The metabolic flux index of heterotrophic CO_2_-fixation, MFI_h-CO2_, can be calculated by the equation at the bottom right, using the determined amount of unlabeled 3PGA (*x* mol) and ^13^C-labbled 3PGA (*y* mol). 3PGA, 3-phosphoglycerate; *AcCoA* acetyl-CoA, *ACE* acetate, *DHAP* dihydroxyacetone phosphate, *E4P* erythrose-4-phosphate, *F6P* frutose-6-phosphate, *FBP* fructose-1,6-biphosphate, *G3P* glyceraldehyde-3-phosphate, *G6P* glucose-6-phosphate, *Glucon6P* gluconate-6-phosphate, *GlyOx* glyoxylate, *ICIT* isocitrate, *MAL* malate, *OAA* oxaloacetate, *PEP* phosphoenolpyruvate, *PRK* phosphoribulokinase, *PYR* pyruvate, *R5P* ribose-5-phosphate, *Ru5P* ribulose-5-phosphate, *Rubisco* ribulose-1,5-bisphosphate carboxylase/oxygenase, *RuBP* ribulose 1,5-bisphosphate, *S7P* sedoheptulose-7-phosphate, *SUC* succinate, *X5P* xylulose-5-phosphate

Herein, we use a heterotrophic CO_2_-fixing *E. coli* strain as a model to elucidate how MFI_h-CO2_ is calculated. The strain was constructed by incorporating two sequential enzymes in the cyanobacterial Calvin cycle, phosphoribulokinase (PRK), and ribulose-1,5-bisphosphate carboxylase/oxygenase (Rubisco) into the central metabolism of *E. coli*. The incorporated CO_2_-fixing bypass pathway starts at ribulose 5-phosphate (Ru5P) in the pentose phosphate pathway of the central metabolism and ends at 3-phosphoglycerate (3PGA) in the glycolysis of the central metabolism (Fig. 1). When the strain is cultured in medium supplemented with ^13^C-labeled sodium bicarbonate, intracellular ^13^CO_2_, either generated by diffusion of the extracellular dissolved ^13^CO_2_ or by the equilibrium of ^13^C-labeled bicarbonate after its active transportation into cell, will be used as the substrate for Rubisco.

As shown in Fig. 1, we assume *a* mole of 3PGA is generated from the central pathway and *b* mole of ^13^CO_2_ is fixed by the Rubisco pathway in a given period of time. Then (*a* + *b*) mole of unlabeled 3PGA and *b* mole of ^13^C-3PGA are generated. At the same period of time, we assume *c* mole of unlabeled 3PGA and *d* mole of ^13^C-3PGA are channeled into the downstream metabolism. It was reported that a small fraction of ^13^C isotope was coupled with all natural ^12^C-containing compounds [21–23]. We then cultivated *E. coli* strains in medium free of any carbon isotope and determined the ratio of ^13^C-3PGA to the unlabeled 3PGA as the basal isotopic level. The ratio was 3.45 % as shown in Additional file 1: Figure S1. We thus assume that 3.45 % of unlabeled 3PGA will convert to its isotopic form. Therefore, the actually detected molar amount of ^13^C-3PGA (*y*) can be calculated by Eq. (1), while the actually detected unlabeled 3PGA (*x*) can be calculated by Eq. (2).

1 $$ y=b+3.45\%\times \left(a+b\right)-d $$

2 $$ x=\left(1-3.45\%\right)\times \left(a+b\right)-c $$

Under a metabolic steady-state, the relationship of *d*, *c*, *x*, and *y* is shown in Eq. (3).

3 $$ d/c=y/x $$

Solution to the equations deduces Eq. (4).

4 $$ {\mathrm{MFI}}_{\mathrm{h}-\mathrm{C}\mathrm{O}2}=b/a=\left(0.97y-0.03x\right)/\left(1.03x-0.97y\right) $$

In this case, only the concentration of ^13^C-labeled and unlabeled 3PGA are required to be determined to calculate the MFI_h-CO2_. Compared with quantification of all intracellular isotropic metabolites to calculate the absolute metabolic flux, we argue that the determination of MFI_h-CO2_ to evaluate the relative metabolic strength of the CO_2_-fixation pathway would be a simple and convenient alternative.

### Construction of a heterotrophic CO_2_-fixing *E. coli*

The Rubisco-encoding genes *rbcL-rbcX-rbcS* from *Synechococcus* sp. PCC7002 and the PRK-encoding gene *prk* from *Synechococcus elongatus* PCC7942 were cloned into pET30a as described previously [24]. The resulted plasmid was designated as pET-RBC-PRK in this study. To verify the function of CO_2_-fixation pathway, Rubisco, and/or PRK were deactivated by introducing site-directed mutations to their conserved catalytic residues, yielding another three plasmids, pET-RBC197-PRK, pET-RBC-PRK2021, and pET-RBC197-PRK2021. Among them, RBC197 indicates a K197M mutation in the conserved catalytic site of the large subunit of Rubisco [25], and PRK2021 carries K20M and S21A mutations in the conserved nucleotide-binding sites of ATP-binding proteins [26].

**Fig. 2:**
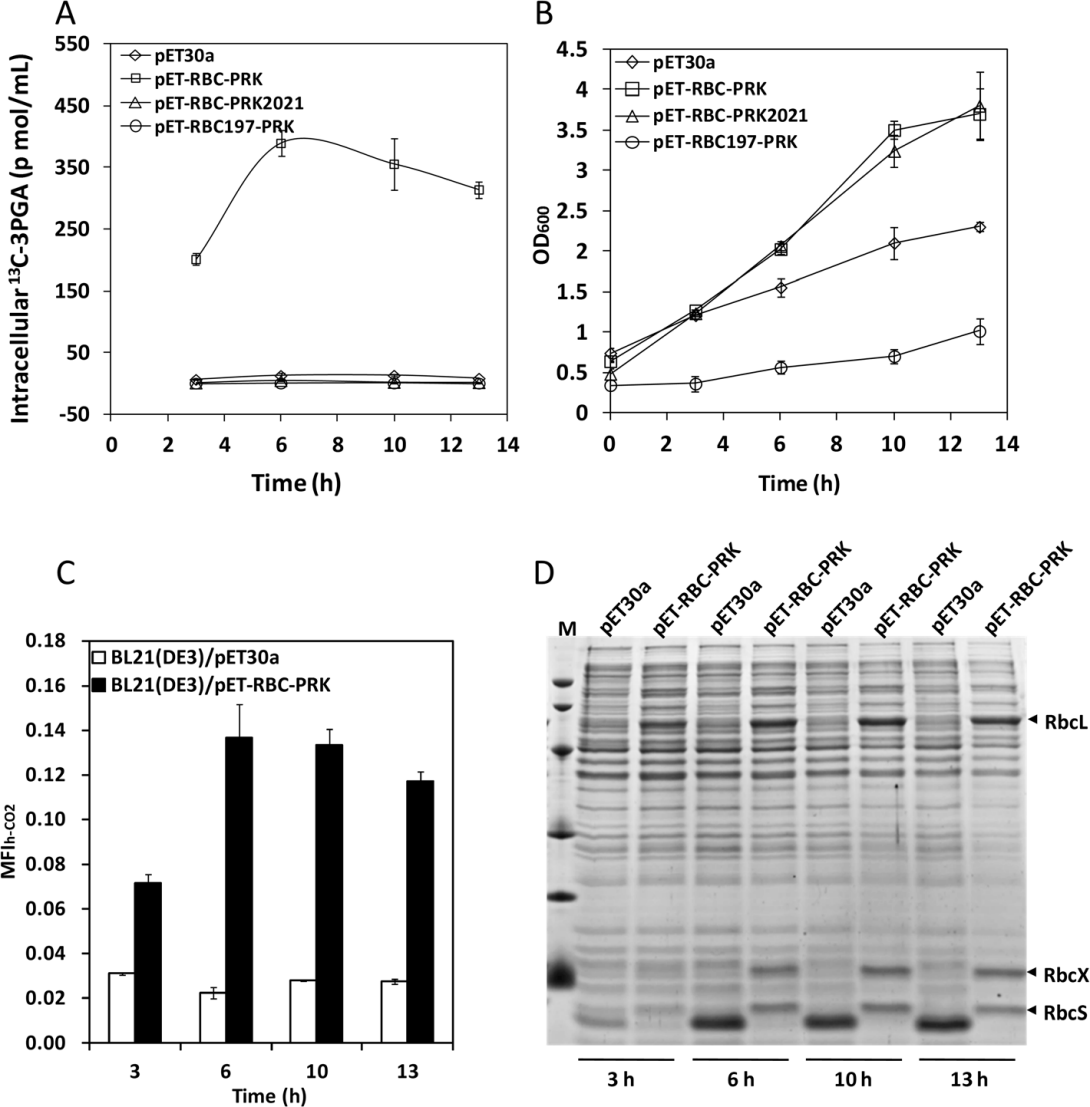
The intracellular ^13^C-3PGA (**a**), cell growth (**b**), MFI_h-CO2_ values (**c**), and soluble protein expression (**d**) of BL21(DE3) strains harboring different plasmids. All strains were 1:100 inoculated into LB medium containing 100 mM NaH^13^CO_3_ and shaken at 37 °C. When the culture reached the mid-log phase (OD_600_ = 0.4–0.6), 0.02 mM IPTG was added to induce Rubisco expression and the induction temperature was reduced to 22 °C (zero point). The PRK-encoding gene under the control of a tryptophan-regulated promoter *trpR*-P_trp_ was leakily expressed in LB medium. RbcL and RbcS are the large and small subunits of Rubisco, which are encoded by *rbcL* and *rbcS* genes, respectively. RbcX is the specific chaperon of Rubisco, which is encoded by the *rbcX* gene. Molecular weight standards from top to bottom are 80, 60, 40, 30, 20, and 12 kDa

Considerable amount of soluble expression of Rubisco under the T7 promoter was observed in strain BL21(DE3) carrying plasmid pET-RBC-PRK upon IPTG induction (Additional file 1: Figure S2). It was reported that the catalytic product of PRK, ribulose 1,5-bisphosphate, could not be metabolized by *E. coli* and thus caused growth arrest to the cell [24, 27]. Retarded cell growth was indeed seen for strain BL21(DE3)/pET-RBC197-PRK with a deactivated Rubisco (Fig. 2b). Hence, the *prk* gene was leakily expressed without induction of its tryptophan-regulated promoter *trpR*-P_trp_ to avoid severe growth inhibition. It is noteworthy that expression of Rubisco and PRK in *E. coli* BL21(DE3) increased cell growth in the late-phase of induction compared with the strain harboring the empty plasmid pET30a without any gene cloned (Fig. 2b). However, this increase appeared not to be the function of enzymes, as similar increases of growth were also seen in the strains transformed with pET-RBC197-PRK containing the deactivated PRK (Fig. 2b) and pET-RBC197-PRK2021 containing both deactivated enzymes (Additional file 1: Figure S3).

As shown in Fig. 2a, a significant increase of the ^13^C-3PGA along with induction time was observed for strain BL21(DE3)/pET-RBC-PRK cultivated with 100 mM NaH^13^CO_3_. Deactivation of either Rubisco or PRK in strain BL21(DE3)/pET-RBC197-PRK or BL21(DE3)/pET-RBC-PRK2021 decreased the ^13^C-3PGA production to the basal level of the control strain BL21(DE3)/pET30a. These results clearly demonstrated that the incorporated Rubisco pathway converted CO_2_ into 3PGA.

The MFI_h-CO2_ values of strain BL21(DE3)/pET-RBC-PRK at different induction times were calculated to evaluate its relative CO_2_ flux (Fig. 2c). For a period of 13 h induction, the MFI_h-CO2_ of the control strain BL21(DE3)/pET30a was below 0.03. Whereas, the MFI_h-CO2_ values of strain BL21(DE3)/pET-RBC-PRK was increased from 0.07 at 3 h to 0.13 at 6 h and then slightly decreased to 0.12 at 13 h. The increase of MFI_h-CO2_ values from 3 to 6 h was associated with the increase of Rubisco expression level (Fig. 2d), suggesting that the increased Rubisco activity contributed to the increased metabolic flux of CO_2_ fixation. When protein expression reached a high level from 6 h onwards, the MFI_h-CO2_ also reached its highest value.

### Identification of the bottleneck of heterotrophic CO_2_ fixation

Rubisco was generally considered as the rate-determining step in the Calvin cycle of autotrophic microbes due to its extremely low catalytic efficiency [28, 29]. For the heterotrophic *E. coli* strain BL21(DE3)/pET-RBC-PRK harboring a partial Calvin cycle, accumulation of RuBP was observed even in the case of leaky-expression of PRK but overexpression of Rubisco. This result suggested that the Rubisco-catalyzed reaction is one of the rate-limiting steps of the CO_2_-fixing bypass pathway in heterotrophic *E. coli* (Additional file 1: Figure S4A). Owing to the difficulty in improving the catalytic activity of Rubisco, we attempted to increase the substrate supply (RuBP or CO_2_) for Rubisco to drive the reaction forward.

**Fig. 3:**
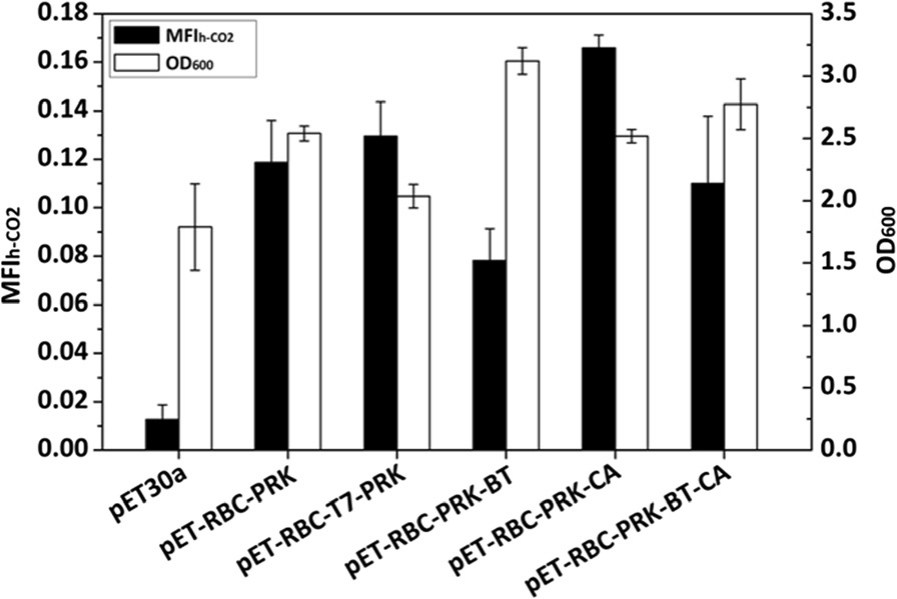
MFI_h-CO2_ values and OD_600_ of BL21(DE3) strains containing the indicated six plasmids. All strains were cultivated in LB medium containing 100 mM NaH^13^CO_3_ and expressed at 22 °C for 10 h in the presence of 0.02 mM IPTG

To increase the supply of RuBP, the weak promoter *trpR*-P_trp_ for PRK expression was replaced by a strong promoter P_T7_, yielding a plasmid pET-RBC-T7-PRK. A significant increase of PRK expression level and an 8.6-fold increase of intracellular RuBP was observed after promoter replacement (Additional file 1: Figure S4). However, no significant difference in the MFI_h-CO2_ value (a *P* value of 0.36 using the Student *T* test) was observed after increasing the intracellular RuBP amount (Fig. 3), indicating that RuBP supply was not the rate-limiting factor.

To increase CO_2_ supply, the unique cyanobacterial carbon concentrating mechanism (CCM) was introduced into *E. coli*. In cyanobacteria, bicarbonate is first transported to plasma membrane by bicarbonate transporter (BT), diffused into caboxysome, and then converted to CO_2_ by carbonic anhydrase (CA) and finally catalyzed by Rubisco therein [30]. To mimic this CCM in *E. coli*, single BT- or CA-encoding gene from *Synechococcus* sp. PCC7002, and their combinations, were respectively introduced into *E. coli*. The *bicA* gene, which encodes a Na^+^-dependent BT with high flux rate [31], was fused with promoter *trpR*-P_trp_ and then inserted into pET-RBC-PRK to generate pET-RBC-PRK-BT. The MFI_h-CO2_ value of strain BL21(DE3)/pET-RBC-PRK-BT exhibited a decrease of 34.1 % compared with that of strain BL21(DE3)/pET-RBC-PRK (Fig. 3). This can be speculated that the increase of intracellular bicarbonate might cause pH variance and possibly affect expression or function of Rubisco or PRK. Moreover, bicarbonate has to be converted to CO_2_ so as to be catalyzed by Rubisco. The equilibrium of bicarbonate and CO_2_ under intracellular condition (e.g., pH 7.5) give the ratio of [HCO_3_^-^]/[CO_2_] to be 14 (the *pK*_a_ of H_2_CO_3_ is 6.35 [32]). The increment of intracellular CO_2_ is thus only 7 % of that of bicarbonate. All these indicated that increasing the intracellular bicarbonate by BT expression was not an effective mean to improve heterotrophic CO_2_ fixation.

The CA-encoding gene (*ccaA*) was fused with a mutated constitutive bacteriophage promoter P_L_-AA [33] and then inserted into pET-RBC-PRK and pET-RBC-PRK-BT. The resultant strains BL21(DE3)/pET-RBC-PRK-CA and BL21(DE3)/pET-RBC-PRK-BT-CA showed MFI_h-CO2_ values of 0.17 and 0.11, respectively, which were 39.8 and 40.7 % higher than those of their respective parent strains without CA insertion (Fig. 3). Overexpression of CA increased the metabolic flux of heterotrophic CO_2_-fixation, indicating that CO_2_ supply is a limiting factor for CO_2_ fixation in *E. coli*.

### Determination of the CO_2_-fixation rate of the heterotrophic *E. coli*

It was reported that *E. coli* metabolized 99 % of the sugar carbon into biomass, CO_2_, and acetate under aerobic condition [34]. However, no obvious fermentation product was detected for the CO_2_-fixing and control *E. coli* strains after 24 h of aerobic cultivation (Additional file 1: Figure S5). The carbon balance calculation of the control strain BL21(DE3)/pET-RBC197-PRK2021 without the ability of CO_2_-fixation also confirmed that the biomass and released CO_2_ accounted for 96 % of the consumed sugar carbon. According to the mass balance of carbon, the fixed CO_2_ of the CO_2_-fixing *E. coli* strain can be calculated by Eq. (5), where all values are in the molar amount of carbon.

5 $$ {\mathrm{C}}_{{\mathrm{in}\ \mathrm{fixed}\ \mathrm{C}\mathrm{O}}_2}={\mathrm{C}}_{{\mathrm{in}\ \mathrm{secreted}\ \mathrm{C}\mathrm{O}}_2}+{\mathrm{C}}_{\mathrm{in}\ \mathrm{biomass}}-0.96\times {\mathrm{C}}_{\mathrm{in}\ \mathrm{consumed}\ \mathrm{sugar}} $$

The specific CO_2_ secretion rate of a given *E. coli* is a constant, which was 11.8 mmol g dry weight^−1^ h^−1^ reported in one literature [35] and 18.6 mmol g dry weight^−1^ h^−1^ in another [34]. Assuming the value is *k*, Eq. (5) can be transformed to Eq. (6).

6 $$ {\mathrm{C}}_{{\mathrm{in}\ \mathrm{fixed}\ \mathrm{C}\mathrm{O}}_2}=\left(k+1\right){\mathrm{C}}_{\mathrm{in}\ \mathrm{biomass}}-0.96\times {\mathrm{C}}_{\mathrm{in}\ \mathrm{consumed}\ \mathrm{xylose}} $$

Mass balance of carbon for the control strain BL21(DE3)/pET-RBC197-PRK2021, which harbored the two deactivated enzymes of the CO_2_-fixing pathway, can generate Eq. (7).

7 $$ 0.96\times {\mathrm{C}}_{\mathrm{in}\ \mathrm{consumed}\ \mathrm{xylose}}\hbox{'}={\mathrm{C}}_{{\mathrm{in}\ \mathrm{secreted}\ \mathrm{C}\mathrm{O}}_2}\hbox{'}+{\mathrm{C}}_{\mathrm{in}\ \mathrm{biomass}}\hbox{'} $$

Assuming the specific CO_2_ secretion rate of the control strain is *k'*, Eq. (7) will be transformed to Eq. (8).

8 $$ 0.96\times {\mathrm{C}}_{\mathrm{in}\ \mathrm{consumed}\ \mathrm{xylose}}\hbox{'}=\left(k\hbox{'}+1\right){\mathrm{C}}_{\mathrm{in}\ \mathrm{biomass}}\hbox{'} $$

Since CO_2_ is mainly generated from the tricarboxylic acid cycle of *E. coli* under aerobic conditions, the incorporated CO_2_-fixing pathway, which is a bypass of the upstream glycolysis, would not affect the specific CO_2_ secretion rate of the strain. Then, under the same cultivation condition, we can assume Eq. (9).

9 $$ k=k\hbox{'} $$

Solution to Eqs. (6), (8), and (9) generates Eq. (10).

10 $$ {\mathrm{C}}_{{\mathrm{in}\ \mathrm{fixed}\ \mathrm{C}\mathrm{O}}_2}=\frac{0.96\times {\mathrm{C}}_{\mathrm{in}\ \mathrm{consumed}\ \mathrm{xylose}}\hbox{'}}{{\mathrm{C}}_{\mathrm{in}\ \mathrm{biomass}}\hbox{'}}{\mathrm{C}}_{\mathrm{in}\ \mathrm{biomass}}-0.96\times {\mathrm{C}}_{\mathrm{in}\ \mathrm{consumed}\ \mathrm{xylose}} $$

Two CO_2_-fixing *E. coli* strains and the control strain were aerobically cultivated in 200 mL of M9 minimal medium supplemented with 10 g L^−1^ xylose in an Erlenmeyer flask. The flask was placed in an air-tight container (10 L) prefilled with 5 % CO_2_ and 95 % air and shaken at room temperature for 24 h. The pH variance, consumed xylose, and generated dry cell weight were determined (Table 1). All cultures maintained a stable pH, with a fluctuation of less than 0.2 unit. Calculation using Eq. (10) indicated that stains BL21(DE3)/pET-RBC-PRK and BL21(DE3)/pET-RBC-PRK-CA were able to fix 13.3 and 19.6 mg CO_2_ L^−1^ h^−1^, respectively. The 47.4 % of increment in the CO_2_-fixation rate after CA expression was similar to the 39.8 % of increment in the MFI_h-CO2_ value, which confirmed that the MFI_h-CO2_ was reliable for evaluating the CO_2_-fixation flux in the heterotrophic *E. coli*. The CO_2_-fixation rates of the heterotrophic *E. coli* strains constructed in this study were compared with those of the natural CO_2_-fixing autotrophic microbes (Table 2). Fourteen autotrophic microbes including microalgae, cyanobacteria, and non-green algae fixed CO_2_ at rates ranging from 10.5 to 147.0 mg CO_2_ L^−1^ h^−1^, with the median value of 21 mg CO_2_ L^−1^ h^−1^. The CO_2_-fixing *E. coli* strains were able to fix CO_2_ at rates of 13.3–19.6 mg CO_2_ L^−1^ h^−1^, which were comparable to the capacity of the autotrophic microbes.

**Table 1:** The pH variance, consumed xylose, generated biomass, and calculated CO_2_-fixation rate of *E. coli* strains after 24 h of aerobic cultivation in 5 % CO_2_

Strain	Initial pH	Final pH^a^	Consumed xylose^a^ (mmol L^−1^)	Biomass^a^ (DCW L^−1^)	CO_2_-fixation rate (mg L^−1^ h^−1^)
BL21(DE3)/pET-RBC-PRK	7.0	6.81 ± 0.06	13.7 ± 1.1	0.82 ± 0.33	13.3 ± 3.2
BL21(DE3)/pET-RBC-PRK-CA	7.0	6.81 ± 0.04	14.8 ± 1.5	0.87 ± 0.29	19.6 ± 4.0
BL21(DE3)/pET-RBC197-PRK2021	7.0	6.87 ± 0.07	29.8 ± 4.7	1.59 ± 0.25	–

^a^The cultivation was independently repeated for three times and the standard deviations were shown after the mean value

**Table 2:** Comparison of the CO_2_-fixation rates of autotrophic and heterotrophic CO_2_-fixing microbes

	Species	CO_2_-fixation rate (mg L^−1^ h^−1^)	Biomass concentration (g DCW L^−1^)	Specific CO_2_-fixation rate^a^ (mg g DCW^−1^ h^−1^)	CO_2_ concentration (%)	Culture condition	References
Autotrophic microbes							
Microalgae	*Chlorella pyrenoidosa* SJTU-2	10.8	1.5	7.3	10	1 L flask with 800 mL WV	[52]
	*Dunaliella tertiolecta* SAD-13.86	11.0	2.1	5.2	10	11 Lfermentor with 8 L WV	[53]
	*Botryococcus braunii* SAG-30.81	21.0	3.1	6.8	10	11 L fermentor with 8 L WV	[53]
	*Scenedesmus obliquus* SJTU-3	12.1	1.8	6.6	10	1 L flask with 800 mL WV	[52]
	*Scenedesmus* sp. NIER-10060	25.5	2.7	9.4	15	Photobioreactor	[54]
	*Chlorella vulgaris* LEB-104	10.5	1.9	5.4	10	11 L fermentor with 8 L WV	[53]
	*Chlorella Vulgaris* NIER-10003	19.2	1.9	10.2	15	Photobioreactor	[54]
	*Chlorella vulgaris*	53.0	5.7	9.3	5	Photobioreactor^c^	[55]
Cyanobacteria	*Spirulina* sp.	17.0^a^	4.8	3.5	6	2 L vertical tubular photobioreactor with 1.8 L WV	[56]
	*Microcystis aeruginosa* NIER-10037	20.4	2.3	8.8	15	Photobioreactor	[54]
	*Microcystis ichthyoblabe* NIER-10040	21.7	2.2	9.8	15	Photobioreactor	[54]
	*Anabaena* sp. ATCC 33047	60.4	2.7	22.4	0.03^b^	Glass bubble column photobioreactor	[57]
	*Aphanothece microscopica*	109.0	5.1	21.4	15	Glass bubble column photobioreactor	[58]
Non-green algae	*Phaeodactylum tricornutum*	147.0	6.2	23.7	40	Photobioreactor	[59]
Heterotrophic microbes							
Bacteria	*E. coli* JB	5.8	6.1^c^	0.95	0.03	3 L fermentor with 1 L WV	[19]
	*E. coli* BL21(DE3)/PET-RBC-PRK	13.3	0.82	16.2	5	1 L flask with 200 mL WV	This study
	*E. coli* BL21(DE3)/PET-RBC-PRK-CA	19.6	0.87	22.5	5	1 L flask with 200 mL WV	This study

*DCW* dry cell weight, *WV* working volume

^a^Calculated by the CO_2_-fixation rate in the unit of mg L^−1^ h^−1^ divided by the biomass concentration in the unit of g DCW L^−1^

^b^Calculated by multiplying the reported OD_600_ (17.63) by our experimentally determined dry cell weight of *E. coli* (0.35 g L^−1^ OD_600_^−1^)

^c^Sequential photobioreactor using recycle water

## Discussion

Recycling CO_2_ directly into fuels or chemicals is a potential approach to reduce carbon emission as well as to resolve energy crisis [6, 7]. The past 5 years have witnessed great success in production of CO_2_-derived molecules that have potential to be used as fuels and chemicals by autotrophic microbes. Quantitative analysis in this study revealed that an engineered heterotrophic *E. coli* could assimilate CO_2_ at a rate comparable to that of the autotrophic cyanobacteria and algae. It is noteworthy that the specific CO_2_-fixation rates of the *E. coli* strains were superior to most of the autotrophic microbes listed in Table 2. Since *E. coli* can easily grow to a high density in fermentors under well-controlled conditions, we believe that heterotrophic microbes might be an alternative candidate for CO_2_ fixation with great potential.

The most striking advantage of using heterotrophic microbes for CO_2_ fixation is their fast growth rates. The doubling times for *E. coli* and yeast are only 20 min [36] and 2 h [37], respectively, whereas those for common cyanobacteria and algae are in the range of 8–44 h [38, 39]. Most autotrophic microbes use photosynthesis to provide energy for CO_2_ assimilation and ultimately biomass accumulation. The theoretical maximum of solar energy conversion efficiency in photosynthesis is only 8–10 % [40], whereas the actual values for several species of cyanobacteria, microalgae, and plants do not exceed 3 % [41]. The low efficiency of photosynthesis can be ascribed to many inherent factors including insufficient absorption of all light wavelengths during light-dependent reactions and low carboxylation activity of Rubisco and existence of energy-consuming photorespiration during light-independent reactions [42]. Although many efforts have been made [43, 44], dramatic increases in photosynthetic efficiency as well as growth rate are still big challenges for autotrophic microbes [44]. However, billions of years of evolution have enabled the heterotrophic microbes to efficiently assimilate the high-energy sugars to generate both carbon backbone and energy at the same time. Therefore, heterotrophic microbes might be a better choice for CO_2_ fixation, since the fixed CO_2_ can be easily joined into the central metabolism and then be efficiently metabolized.

For the current version of the CO_2_-fixing *E. coli* strain constructed in this study, CO_2_ was fixed at the expense of sugar consumption because all energy required for CO_2_ fixation comes from sugar. However, it is not unbelievable that CO_2_ fixation can occur without sugar consumption in heterotrophic microbes once energy can be supplied from other sources. The pioneer work by Liao’s group has demonstrated that electricity can be used as the sole energy to convert CO_2_ to higher alcohols in *Ralstonia eutropha* [8], opening the door of employing other energy forms for CO_2_ fixation.

There is no doubt that improving the carboxylation activity of Rubisco is the ultimate way to increase the efficiency of CO_2_ fixation in both autotrophic and heterotrophic microbes. However, decades of Rubisco engineering gained limited success [24, 45]. In this work, the difficulty of Rubisco in access to CO_2_ was found to be another limiting factor of heterotrophic CO_2_ fixation. Expression of the CA from *Synechococcus* sp. PCC7002 under a weak constitutive promoter increased the *E. coli* CO_2_-fixation rate by 47.4 %. It is thus suggested that screening of the CA gene and optimization of its expression might be feasible ways to further improve the heterotrophic CO_2_-fixation rate. CA, which catalyzes the reversible interconversion of CO_2_ and HCO_3_^−^, is widely existed in animals, plants, archaebacteria, and eubacteria, and plays an important role in many physiological functions [46]. Although some CAs prefer the direction of CO_2_ hydration, the carboxysomal CAs in cyanobacteria and some chemoautotrophic bacteria favor the direction of HCO_3_^−^ dehydration. To date, two forms of carboxysomal CAs (α and β), which are encoded by three types of genes with distinct sequences and structures (*CsoSCA* for α-CA and *CcaA* and *CcmM* for β-CA), were reported [47, 48]. The selected CA-encoding gene from *Synechococcus* sp. PCC7002 in this study was the *CcaA* gene. Whether the other two types of CA-encoding genes can be expressed in *E. coli* and whether their expression can increase the heterotrophic CO_2_-fixation rate are now under investigation by our group. Moreover, a stronger inducible promoter might be employed to enhance the CA expression in a controllable way to further improve the CO_2_ supply.

As a compensation for the low carboxylation activity of Rubisco, some autotrophic microbes have evolved some physical barriers (e.g., the semi-permeable caboxysome in cyanobacteria and the bundle sheath cells in C4 plants) to concentrate CO_2_ around Rubisco. Inspired by these, we suppose that constraining CO_2_ and the CO_2_-fixing enzyme in a microcompartment (e.g., reconstruction of the caboxysome in *E. coli* [49]) or recruiting the CO_2_-producing and CO_2_-fixing enzymes in a protein/RNA scaffold in *E. coli* might be an alternative way to further improve its CO_2_-fixation rate.

## Conclusions

In this study, quantitative analysis approaches have been developed for CO_2_ fixation in heterotrophic microbes. The difficulty in access to CO_2_ was found to be a limiting factor for heterotrophic CO_2_ fixation. An *E. coli* strain capable of fixing CO_2_ at a rate of 19.6 mg CO_2_ L^−1^ h^−1^ or 22.5 mg CO_2_ g DCW^−1^ h^−1^ was constructed by incorporation of partial cyanobacterial Calvin cycle and carbon concentrating mechanism. This work demonstrated that CO_2_ fixation by the engineered heterotrophic *E. coli* can be as effective as the natural autotrophic cyanobacteria and algae, showing great potential of heterotrophic CO_2_ fixation.

## Methods

### Plasmids construction

All plasmids were constructed based on pET30a (Additional file 1: Table S1) and transformed to *E. coli* BL21 (DE3) for protein expression. The primers used are listed in Additional file 1: Table S2.

### Isotropic assay for CO_2_-fixation efficiency

A fresh single colony of the strain was inoculated into LB medium containing 50 ng μL^−1^ kanamycin and cultured overnight at 37 °C. An aliquot of 100 μL of the overnight culture was inoculated into 40 mL fresh LB medium containing 50 ng μL^−1^ kanamycin, 100 mM hydroxyethylpiperazine ethanesulfonic acid (HEPES), and 100 mM NaH^13^CO_3_ (Sigma). The culture was shaken at 37 °C until its OD_600_ reached 0.4–0.6. Then the temperature was reduced to 22 °C for maximal protein expression. At intervals, 3 OD_600_ of cells were harvested for SDS-PAGE and 8 mL of cells for intracellular metabolites extraction.

For SDS-PAGE, 3 OD_600_ of cells were resuspended in 1 mL buffer (100 mM HEPES, pH 8.0, 20 mM MgCl_2_, 10 mM KCl, 1 mM EDTA) and sonicated. A 7 μL aliquot of the supernatant fraction (soluble protein) was subjected to SDS-PAGE (12 % w/v).

For intracellular metabolites extraction, all experiments were done on ice. At first, 10 mL of culture were rapidly centrifuged and washed in 10 mL cold (−20 °C) aqueous methanol solution (60 %, *v*/*v*) to quench cell metabolism as soon as possible. The suspension was clarified at −20 °C for 5 min at 20,000 *g*. The cell pellet was resuspended in 80 μL cold (−20 °C) aqueous methanol solution (60 %, *v*/*v*). After addition of 100 μL of 0.3 M KOH (dissolved in 25 % ethanol), the mixture was stored at −80 °C for more than 2 h to break the cell wall. The alkaline mixture was thawed on ice and neutralized by adding 2 μL of glacial acetic acid. Then the sample was centrifuged at −20 °C for 10 min at 20,000 *g*. The supernatant was stored at −80 °C before LC-MS/MS detection [50].

### LC-MS/MS detection

Agilent 6460 series LC-MS/MS system equipped with a HPLC system and a triple-quadrupole Mass Spectrometer were used. All samples were separated by the reversed phase ion pair high performance liquid chromatography with Agilent XDC18 column (5uM, 150 mm × 4.6 mm). The negative ion and selected multiple reactions monitoring (MRM) mode were used for MS detection. Di-*n*-butylammonium acetate (DBAA) was used as the volatile ion pair reagent. DBAA and standard metabolites (3PGA and RuBP) were purchased from Sigma-Aldrich. Methanol was purchased from Fisher Scientific [51]. The mobile phase was the mixture of solution A (water with 5 mM DBAA) and solution B (methanol with 5 mM DBAA) prepared at the gradient shown in Additional file 1: Table S3. The flow rate was 0.6 mL min^−1^. The injection volume was 50 μL and the column temperature was 40 °C.

The settings for MS were as follows: gas temperature, 350 °C; gas flow, 8 L min^−1^; nebulizer, 38 psi; sheath gas temperature, 350 °C; sheath gas flow, 9 L min^−1^; capillary, −3500 V; nozzle voltage, 500 V. The dwell time was set at 200 ms. The MRM parameters were optimized by the standards, and the detailed values for Q1 (m/z of precursor ion), Q3 (m/z of product ion), fragmentor, and collision energy (CE) were listed in Additional file 1: Table S4. All metabolites were quantified by their standard curves.

### HPLC detection

The concentrations of xylose in medium before and after cultivation were determined using an Agilent 1200 high performance liquid chromatography (Agilent Technologies, Santa Clara, CA, USA) with a refractive index (RI) detector. An Aminex HPX-87 H organic acid analysis column (7.8 × 300 mm) (Bio-Rad Laboratories, Inc, CA, USA) was maintained at 15 °C with 0.05 mM sulfuric acid as mobile phase. The injection volume was 10 μL and the flow rate was 0.5 mL min^−1^.

## Additional file

Additional file 1: Tables S1–S5 and Figures S1–S5.
**Table S1.** Plasmids used in this study. **Table S2.** Oligonucleotides used in this study. **Table S3.** Gradient profile of LC-MS/MS. **Table S4.** Optimized parameters of MRM. **Table S5.** Carbon balance of strain BL21(DE3)/pET-RBC197-PRK2021 after 20 h of aerobic cultivation in M9/xylose medium. **Figure S1.** Determination of the basal level of ^13^C-3PGA which was naturally converted by the unlabeled 3PGA. **Figure S2.** Soluble Rubisco expression of BL21(DE3) strains harboring different plasmids. **Figure S3.** Cell growth for strains BL21(DE3)/pET30a, BL21(DE3)/pET-RBC-PRK, and BL21(DE3)/pET-RBC197-PRK2021. **Figure S4.** The amount of intracellular RuBP (A) and soluble proteins (B) for BL21(DE3) strains harboring plasmids pET30a, pET-RBC-PRK, pET-RBC197-PRK, and pET-RBC-T7-PRK, respectively. **Figure S5.** HPLC detection of fermentation products of different strains at 0 h and 24 h of cultivation.

## Abbreviations

Rubisco: ribulose-1,5-bisphosphate carboxylase/oxygenase
PRK: phosphoribulokinase
Ru5P: ribulose 5-phosphate
3PGA: 3-phosphoglycerate
BT: bicarbonate transporter
CA: carbonic anhydrase
MFI_h-CO2_: metabolic flux index of heterotrophic CO_2_ fixation
DCW: dry cell weight
LC-MS/MS: liquid chromatography–mass spectrometry/mass spectrometry
MRM: multiple reactions monitoring
DBAA: Di-*n*-butylammonium acetate
HPLC: high performance liquid chromatography
