# Supplementary material for: Quantitative analysis of an engineered CO2-fixing Escherichia coli reveals great potential of heterotrophic CO2 fixation
Source: Biotechnol Biofuels. 2015 Jun 18;8:86. doi: 10.1186/s13068-015-0268-1 (PMC4475311; doi:10.1186/s13068-015-0268-1)
Supplement: Additional file 1: Tables S1–S5 and Figures S1–S5. — Table S1. Plasmids used in this study. Table S2. Oligonucleotides used in this study. Table S3. Gradient profile of LC-MS/MS. Table S4. Optimized parameters of MRM. Table S5. Carbon balance of strain BL21(DE3)/pET-RBC197-PRK2021 after 20 h of aerobic cultivation in M9/xylose medium. Figure S1. Determination of the basal level of 13C-3PGA which was naturally converted by the unlabeled 3PGA. Figure S2. Soluble Rubisco expression of BL21(DE3) strains harboring different plasmids. Figure S3. Cell growth for strains BL21(DE3)/pET30a, BL21(DE3)/pET-RBC-PRK, and BL21(DE3)/pET-RBC197-PRK2021. Figure S4. The amount of intracellular RuBP (A) and soluble proteins (B) for BL21(DE3) strains harboring plasmids pET30a, pET-RBC-PRK, pET-RBC197-PRK, and pET-RBC-T7-PRK, respectively. Figure S5. HPLC detection of fermentation products of different strains at 0 h and 24 h of cultivation. [file 13068_2015_268_MOESM1_ESM.docx]

**Additional file**

**Quantitative analysis of an engineered CO_2_-fixing *Escherichia coli* reveals great potential of heterotrophic CO_2_ fixation**

Fuyu Gong, Guoxia Liu, Xiaoyun Zhai, Jie Zhou, Zhen Cai, Yin Li

**Table S1 Plasmids used in this study**

| Name | Description | Source |
| --- | --- | --- |
| pET30a | Km^50^ | Lab storage |
| pET-RBC-PRK | Inserting Rubisco encoding genes (*rbcL*-*rbcX*-*rbcS*) with two P_T7_ promoters each at the upstream of *rbcL* and *rbcX* into the NdeI/XhoI sites; inserting PRK encoding gene (*prk*) and the upsteam *trpR*-P_trp_ promoter into the *Fsp*I/*Psh*AI sites | [24] |
| pET-RBC197-PRKK | Derived from pET-RBC-PRK; inactivating Rubisco by a K197M mutation at *rbcL* | [24] |
| pET-RBC-PRK2021 | Derived from pET-RBC-PRK; inactivating PRK by two K20M and S21A mutations | [24] |
| pET-RBC197-PRK2021 | Derived from pET-RBC-PRK, containing both inactive Rubisco and PRK mutants | This work |
| pET-RBC-T7-PRK | Derived from pET-RBC-PRK; changing PRK promoter to P_T7_ | This work |
| pET-RBC-PRK-BT | Derived from pET-RBC-PRK, inserting bicarbonate transporter (BT) encoding gene (*bicA*) and a *trpR*-P_trp_ promoter into the *Bst*Z17I site | This work |
| pET-RBC-PRK-CA | Derived from pET-RBC-PRK, inserting carbonic anhydride (CA) encoding gene (*ccaA*) and a P_L_-AA promoter into the *Bst*Z17I site | This work |
| pET-RBC-PRK-CA-BT | Derived from pET-RBC-PRK-CA, inserting bicarbonate transporter (BT) encoding gene (*bicA*) and a *trpR*-P_trp_ promoter into the *Bst*Z17I site | This work |

**Table S2 Oligonucleotides used in this study**

| Name | Sequence (5’—3’) | Purpose |
| --- | --- | --- |
| 121- trpR-trp-F | CCGGTATACTCCCGGCCGATTTGCGGCCGCCATGCATGCTGAAATTACGGGTATTTGTA | Cloning of *trpR*-P_trp_ promoter |
| 122- trpR-trp-R | TCCCCTAGGGCCGGCATGGCCATTGTCGATACCCTTTTTAC |  |
| 127-bicA-F | TCCCCTAGGATGCAGATAACCAACAAAATTCAC | Cloning of *bicA* |
| 115- bicA-R | CCGGTATACACCAGGCTTGAGTATAGCCTGGTTAACCCATCTCTGAACTGGGAGCCGTA |  |
| 128-PPL-AA-F1 | CCGGTATACTCCCGGCCGATTTGCGGCCGCCATGCATGCCAATTCCGACGTCTAAGAAA | Cloning of P_L_-AA promoter for overlapping with CA |
| 129-PPL-AA-R1 | ATACGCCTATTTTTATAGGTTAATGTCATAATAATAATGGTTTCTTAGACGTCGGAATT |  |
| 130-PPL-AA-F2 | CCTATAAAAATAGGCGTATCACGAGGCCCTTCCGTCTTCACCTCGAGTCCCTATCAGTG |  |
| 131-PPL-AA-R2 | GCTCAGTATTTCTATCACTGATAGGGATGTCAATCTCTATCACTGATAGGGACTCGAGG |  |
| 132-PPL-AA-F3 | AGTGATAGAAATACTGAGCACATCAGCAGGACGCACTGACCCAATAATTTTGTTTAACT | Cloning of CA for overlapping with P_L_-AA promoter |
| 140-7002CA-R | CCGGTATACACCAGGCTTGAGTATAGCCTGGCTAACCACTCACAGAGAAGGTATCAG |  |
| T7-PRK-F | CCGTGCGCATAATACGACTCACTATAGGG | Cloning of P_T7_ promoter for overlapping with PRK |
| T7-PRK-overlap-R | GATCTGGCTTGCTCATATGTATATCTCC |  |
| T7-PRK-overlap-F | GGAGATATACATATGAGCAAGCCAGATCGTGTTG | Cloning of PRK for overlapping with P_T7_ promote |
| PRK-R | ATATTTGACTATCGTCACCAGGCTTGAGTATAGCCTGGCTAGACGCTAGCGGCGACGGG |  |
| 62-seq- rbcL -F | TCGATCTCGATCCCGCGAAAT | Sequencing *rbcL* |
| 63-seq-rbcL-R | ACCACCAAACTGGAGGCAGGAATCG | Sequencing *rbcL* |
| 64-seq-rbcL-rbcX-F | ACCCAAGACTACGCTTCTCTCCCCG | Sequencing *rbcL* and *rbcX* |
| 65-seq-rbcS-R | GCTCAGCGGTGGCAGCAGCCA | Sequencing *rbcS* |
| Seq-prk-R | ATCTCGACCGATGCCCTTGAG | Sequencing *prk* |
| Seq-prk-F | TTTTATCGCAACTCTCTACTG | Sequencing *prk* |
| 119-FspI-seq-F | AGACGTGGCGCATCAGGCATCGTGCACCGAATGCCGGATG | Sequencing P_L_-AA-CA and *trpR*-P_trp_-*bicA* |
| 120-Bstz17I-seq-R | CCTGATGCGGTATTTTCTCCTTACGCATCTGTGCGGTATT | Sequencing P_L_-AA-CA and *trpR*-P_trp_-*bicA* |
| 142-typ-750s-seq-R | CTTGAGCGACACGAATTATGCAGTGATTTA | Sequencing *typ* |
| 144-typ-241s-seq-F: | CAACAGCTCT TCGACAATACGCACGCGAGT | Sequencing *typ* |
| 145-bicA-150s-seq-R | TCCCCCAAAGAGGGCGGCAAAGAAGCCCAC | Sequencing *bicA* |
| 146-typ-400s-seq-R | TATTATGGCCCAACAATCACCCTATTCAGC | Sequencing *typ* |

**Table S3 Gradient profile of LC-MS/MS**

| Step | Total time (min) | Solution A (vol. %) | Solution B (vol. %) |
| --- | --- | --- | --- |
| 1 | 0 | 100 | 0 |
| 2 | 0.33 | 100 | 0 |
| 3 | 10.33 | 90 | 10 |
| 4 | 20.33 | 40 | 60 |
| 5 | 22.00 | 0 | 100 |
| 6 | 25.00 | 0 | 100 |
| 7 | 25.5 | 100 | 0 |
| 8 | 30 | 100 | 0 |

**Table S4 Optimized parameters of MRM**

| Compound | Q1 (m/z) | Q3 (m/z) | Main product ion | Fragmentor (V) | CE (v) |
| --- | --- | --- | --- | --- | --- |
| 3PGA | 184.9 | 96.9 | [H_2_PO_4_]^-^ | 75 | 10 |
| ^13^C-3PGA | 185.9 | 96.9 | [H_2_PO_4_]^-^ | 75 | 10 |
| RuBP | 308.9 | 78.9 | [PO_3_]^-^ | 85 | 80 |

3PGA: 3-phosphoglycerate;

RuBP: ribulose 1,5-bisphosphate;

**Table S5 Carbon balance of strain BL21(DE3)/pET-RBC197-PRK2021 after 20 h of aerobic cultivation in M9/xylose medium**

| **Carbon** | **Amount** |
| --- | --- |
| Consumed carbon (mmol L^-1^) ^a^ | 107 |
| Consumed xylose (mmol L^-1^) ^b^ | 21.40 ± 1.55 |
| Produced carbon ^c^ | 102.71 |
| Biomass (DCW L^-1^) | 1.11 ± 0.03 |
| CO_2_ release (mmol L^-1^) ^d^ | 58.31 ± 4.33 |
| Carbon balance ^e^ | 96.0% |

^a^ The consumed carbon was calculated by multiplying the molar amount of consumed xylose by five.

^b^ The xylose concentrations in the culture supernatants at 0 h and 20 h of cultivation were determined by HPLC. The consumed xylose was the difference between the two xylose concentrations.

^c^ The produced carbon was the sum of the molar amounts of carbon in biomass and CO_2_. The mass of carbon in biomass was calculated by multiplying the mass of biomass by a coefficient of 0.48, which is the experimentally determined percentage of carbon in dry cell of *E. coli* (Bratbak G, Dundas I: Bacterial dry matter content and biomass estimations. Applied and environmental microbiology 1984, 48:755-757).

^d^ The percentages of gaseous CO_2_ in the headspace of the air-tight container at 0 h and 20 h of cultivation were taken by a syringe for GC detection. The molar amount of gaseous CO_2_ was calculated by the equation of state of ideal gas ($\mathrm{Gaseous}\mathrm{CO}_{2} (mol)=\frac{P_{CO2}V}{\mathrm{RT}}$), where the partial pressure of CO_2_, P_CO2_, was calculated by multiplying the CO_2_ percentages by 101325 Pa; the volume of the container was 10 L; the gas constant R was 8.31×10^3^ pa L mol^-1^ K^-1^, and the thermodynamic temperature T was 298 K.

^e^ The carbon balance was calculated by dividing the amount of produced carbon by that of the consumed carbon.


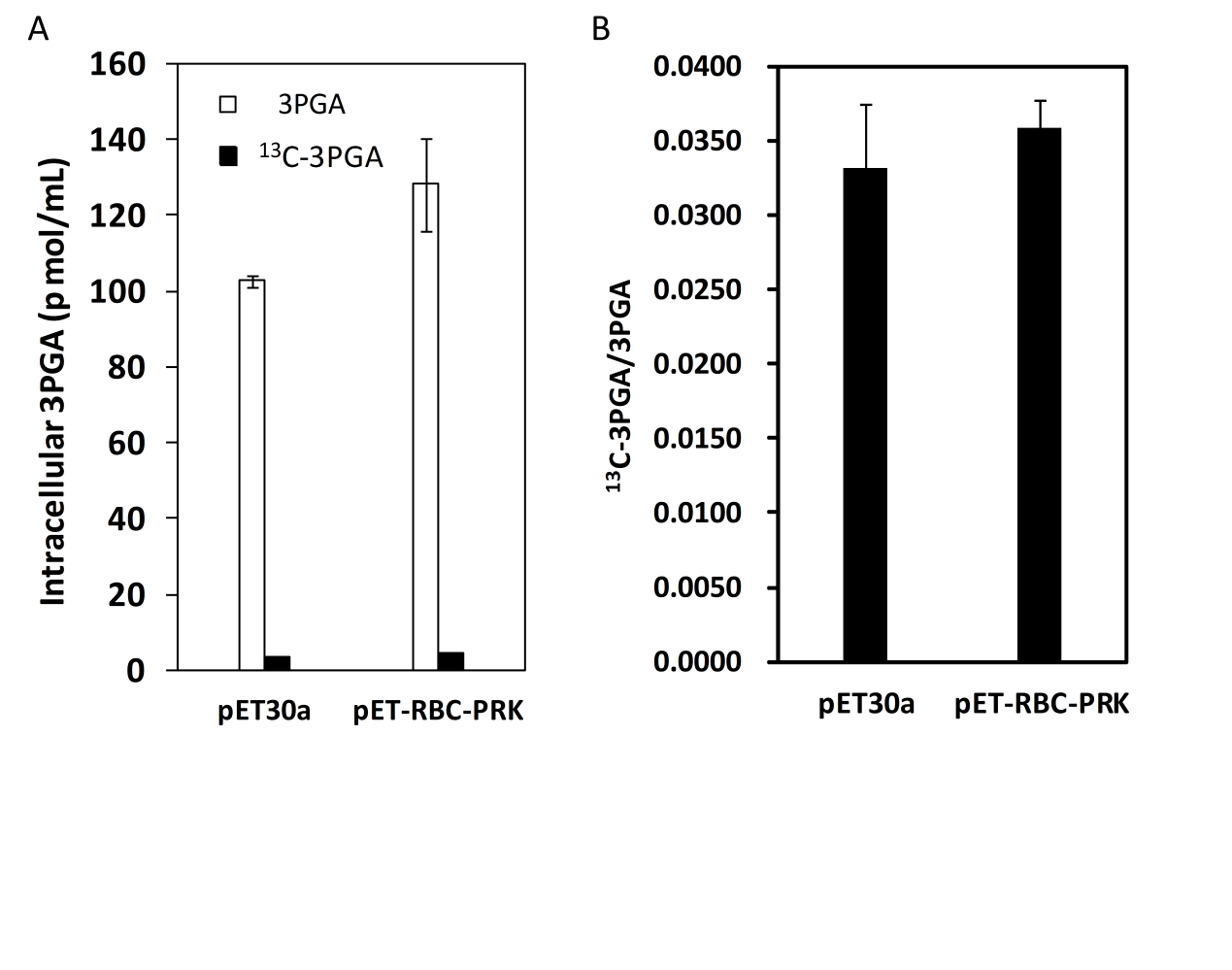


**Figure S1 Determination of the basal level of ^13^C-3PGA which was naturally converted by the unlabeled 3PGA. (A)** The amount of intracellular unlabeled 3PGA and ^13^C-labeled 3PGA for BL21(DE3) strains harboring pET30a and pET-RBC-PRK cultivated with 100 mM unlabeled NaHCO_3_. The ratio of ^13^C-3PGA to unlabeled 3PGA was calculated in **(B)**. The ratios for the two strains are 3.32% and 3.59%, respectively, with the average value of 3.45%.

**
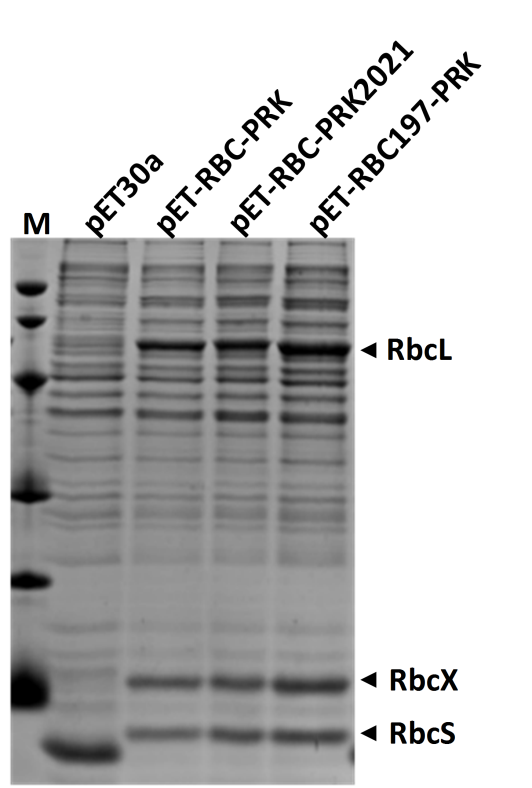
**

**Figure S2 Soluble Rubisco expression of BL21(DE3) strains harboring different plasmids.** All strains were 1:100 inoculated into LB medium containing 100 mM NaH^13^CO_3_ and shaken at 37^o^C. When the culture reached the mid-log phase (OD_600_=0.4-0.6), 0.02 mM IPTG was added to induce Rubisco expression and the induction temperature was reduced to 22^o^C for 10 h. RbcL and RbcS are the large and small subunits of Rubisco, which are encoded by *rbcL* and *rbcS* genes, respectively. RbcX is the specific chaperon of Rubisco, which is encoded by the *rbcX* gene. Molecular weight standards from top to bottom are 80, 60, 40, 30, 20, and 12 kDa.


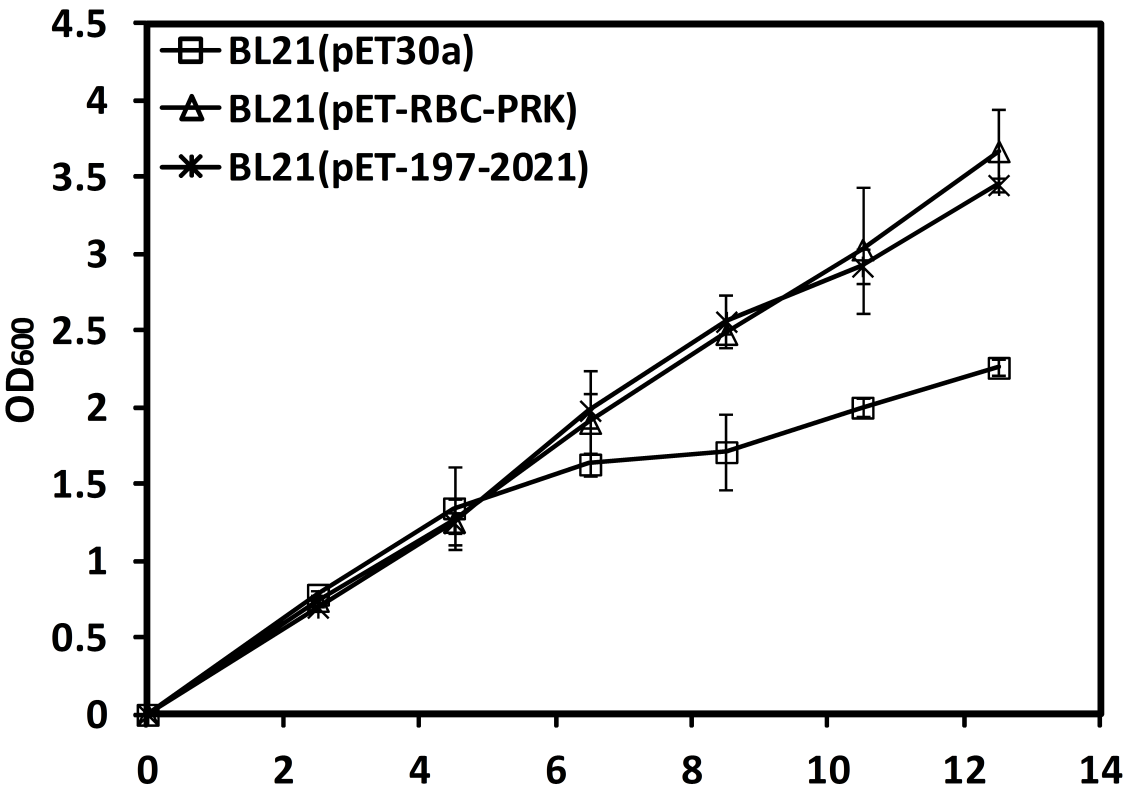


**Figure S3 Cell growth of strains BL21(DE3)/pET30a, BL21(DE3)/pET-RBC-PRK, and BL21(DE3)/pET-RBC197-PRK2021**. All strains were cultivated in LB medium with 50 ng μL^-1^ kanamycin, 100 mM HEPES, and 100 mM NaH^13^CO_3_.


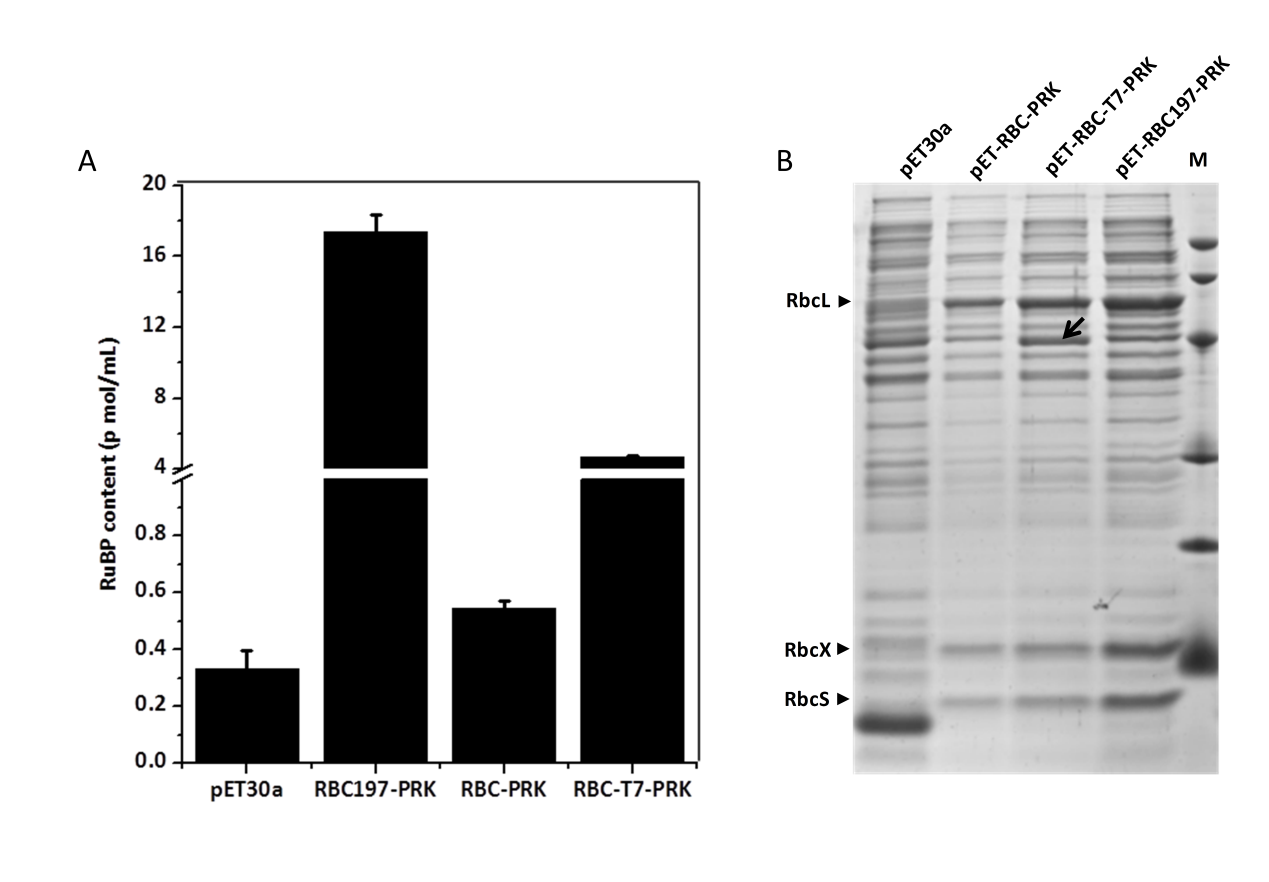


**Figure S4** **The amount of intracellular RuBP (A) and soluble proteins (B) for BL21(DE3) strains harboring plasmids pET30a, pET-RBC-PRK, pET-RBC197-PRK, and pET-RBC-T7-PRK, respectively.** Molecular weight standards from top to bottom are 80, 60, 40, 30, 20, and 12 kDa. The PRK was indicated by an arrow.


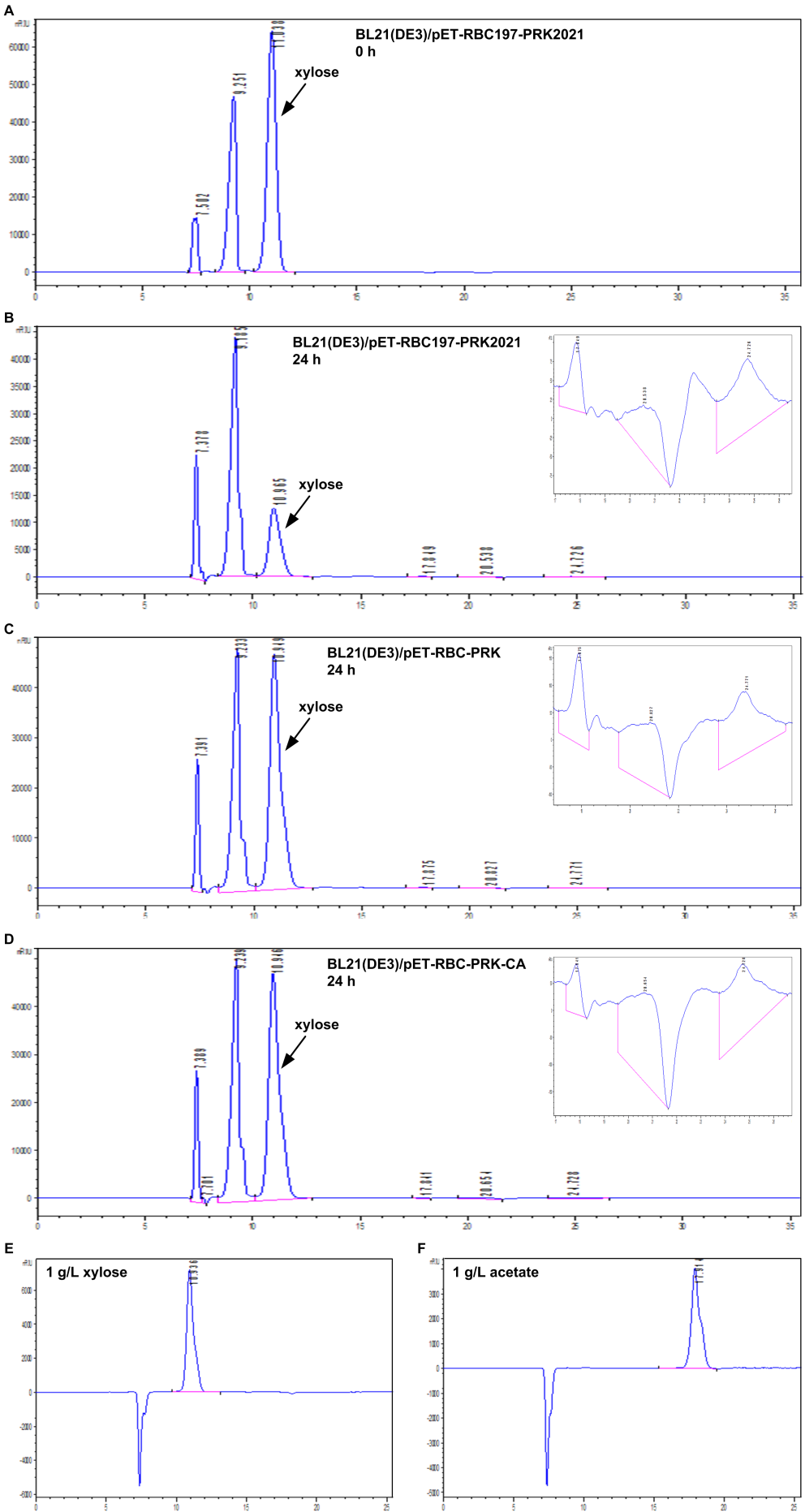


**Figure S5** **HPLC detection of the fermentation products of different strains at 0 h and 24 h of cultivation.** All strains were cultivated in 200 mL of M9 medium with 10 g L^-1^ xylose in an air-tight container (10 L) prefilled with 5% CO_2_ and 95% air and shaken at room temperature for 24 h. Enlarged graphs of the three putative peaks between retention times of 17-27 min in panels (B)-(D) were shown in the upper-right corners of each panel.
